# The role, relevance and management of immune exhaustion in bovine infectious diseases

**DOI:** 10.1016/j.heliyon.2024.e28663

**Published:** 2024-03-27

**Authors:** Shalini Sharma, Naveen Kumar, Barry T. Rouse, Khushbu Sharma, Kundan Kumar Chaubey, ShoorVir Singh, Praveen Kumar, Pradeep Kumar

**Affiliations:** a Department of Veterinary Physiology and Biochemistry, Lala Lajpat Rai University of Veterinary and Animal Sciences, Hisar, 125004, Haryana, India; b National Center for Veterinary Type Cultures, ICAR-NRC on Equines, Sirsa Road, Hisar, Haryana, 125001, India; c College of Veterinary Medicine, University of Tennessee, Knoxville, TN, 37996-0845, USA; d Department of Biotechnology, School of Basic and Applied Sciences, Sanskriti University, Mathura, Uttar Pradesh, 281 401, India; e Department of Bio-technology, GLA University, Post-Chaumuhan, Dist. Mathura, Uttar Pradesh, 281 406, India; f Department of Veterinary Medicine, Lala Lajpat Rai University of Veterinary and Animal Sciences, Hisar, 125004, Haryana, India

**Keywords:** Immune, Exhaustion, Bovine, Immuno-inhibitory receptors, Cytokines, T cells-CD4 and CD8, B cells

## Abstract

Immune exhaustion is a state of immune cell dysfunction that occurs most commonly following chronic exposure to an antigen which persists after the immune response fails to remove it. Exhaustion has been studied most thoroughly with several cancers, but has also been observed in several chronic infectious diseases. The topic has mainly been studied with CD8^+^ T cells, but it can also occur with CD4^+^ T cells and other immune cell types too. Exhaustion is characterized by a hierarchical loss of effector cell functions, up-regulation of immuno-inhibitory receptors, disruption of metabolic activities, and altered chromatin landscapes. Exhaustion has received minimal attention so far in diseases of veterinary significance and this review's purpose is to describe examples where immune exhaustion is occurring in several bovine disease situations. We also describe methodology to evaluate immune exhaustion as well as the prospects of controlling exhaustion and achieving a more suitable outcome of therapy in some chronic disease scenarios.

## 1 Immune exhaustion: An introduction

Following an acute infection, naive T cells are activated and undergo proliferation, metabolic reprogramming and develop effector T cell responses which includes cytokine secretion, altered tissue homing and other events that subscribe to terminating the infection.After effector responses reach their peak, the antigen is eliminated, the inflammation resolves, and the majority of activated effector T cells die. However, a small fraction survives and matures into a pool of memory T cells which upon antigen re-exposure rapidly proliferate and reactivate their effector functions and the infection is resolved with greater efficiency. The scenario can be quite different with infections that are resistant to immune control and persist to set up a chronic infection. In such infections, continuous exposure to antigen can result in the ability of T cells to gradually lose some and eventually all of their effector functions and express a number of inhibitory molecules [1]**.** Similar to chronic infections, persistent antigenic stimulation under sub-optimal conditions has been shown to result in induction of T-cell exhaustion as has been mainly studied in cancers. Immune exhaustion refers to a state of cellular immune dysfunction (more importantly, the absence of a conventional effector response**)** [2]**.**

Of high relevance is the fact that the exhausted cells express many inhibitory molecules which when blocked by specific antibodies can result in the exhaustion state being fully or partially reversed and the return of effective immune function. The initial inhibitory molecule discovered was called CTLA-4 (cytotoxic T-lymphocyte–associated antigen 4) and its discoverer (James Allison) being awarded the Nobel Prize in 2018. Other inhibitory proteins which are up-regulated by exhausted cells, include programmed cell death protein-1 (PD-1), T cell immunoglobulin and mucin protein 3 (TIM-3) and lymphocyte-activation gene 3 (LAG-3). Such exhausted cells are less activated, have compromised recall potential upon antigenic exposure, express diminished proliferative potential, demonstrate hierarchical loss of effector cytokine production, more prone to apoptosis [2], change expression and usage of pivotal transcription factors and some metabolic pathways, and fail to develop quiescence and memory T cell homeostasis, which is no longer dependent on antigen [3]. Derangements in metabolism coincide with the disarmament of CD8^+^ T-cell responses leading to exhaustion in cases of chronic infection or cancers [4,5]. We have now begun to explore the mechanistic insights into the development of T cell exhaustion in chronic viral infection models that includes ruminants.

Most investigations to date have studied exhaustion in T cells, especially CD8^+^ T cells [6], but other cell types that include CD4 [7] and B cells and conceivably some members involved in innate immunity may also become immunosuppressed/exhausted [8,9]. Immunosuppression is temporary or permanent immune dysfunction in response to insults to the immune system (Dohms and Saif 1984). Extensive studies have been performed in experimental murine systems to define the mechanisms involved in exhaustion, the relevance of targeting one or more inhibitory systems during therapy and the involvement of cells in addition to CD8^+^ T cells that include members of the innate immune system that usually do not manifest immunological memory. As of the present there has been very minimal exploration of the relevance of immune exhaustion in other than murine or human diseases, yet it seems likely that several domestic animal cancers and chronic infectious diseases could involve exhausted immune cells and which may be reversed by appropriate therapy. In this review we have described what we know and more importantly what we need to know as regards the role of exhausted immune cells during chronic diseases that affect ruminant species. We describe how to detect exhaustion in bovine systems and demonstrate several instances where exhaustion occurs. We also discuss how exhaustion could be managed particularly in a situation where therapy costs must be kept to a minimum.

## 2 T cell exhaustion reported in bovine infectious diseases

**Fig. 1:**
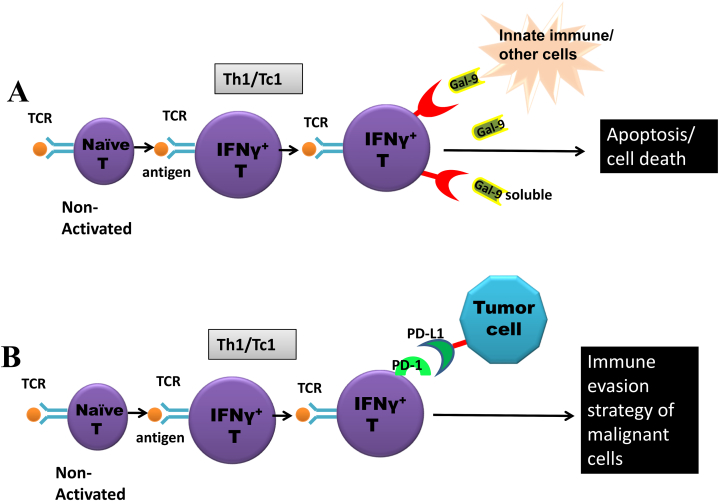
A Upon activation naive cells become activated effector T cell (T helperTh1/T cytotoxic Tc1). Activated effector T cells up-regulates TIM-3 (an immuno-inhibitory receptor) which upon binding to its ligand galectin 9 (expressed on innate cells and other immune cells) or available as soluble form, result in apoptosis of TIM-3 bearing effector T cells. This results in dampening of the immune response which might otherwise result in immuno-pathology due to excessive cytokine production by the immune effector cells. Remaining cells down regulates TIM-3 after peak of an immune response. Thus TIM-3-Galectin 9 is an immunoregulatory pathway. Fig. 1B High PD-L1 expression is expressed in the thymus and on dendritic cells, where the PD-L1/PD-1 interaction prevents the proliferation and differentiation of naïve T cells. PD-1-PD-L1 pathway is also involved in immune exhaustion. Persistent up-regulation of PD-1 is expressed on tumour-infiltrating lymphocytes, where PD-L1 expression is exploited by malignant cells to avoid immune destruction (an immune evasion strategy by tumor cells).

a. Bovine leukemia virus:

PD-1 is involved in the reduction of immune responses via alteration of T-cell activity, promoting programmed cell death of antigen-specific T cells and restraining that of regulatory T cells [10]. The programmed cell death ligand 1 (PD-L1) is a *trans*-membrane protein that is a ligand for PD-1.The PD-1-PD-L1 interaction (Fig. 1B) restricts the proliferation of PD-1expressing cells, inhibits their cytokine production and promotes apoptosis [10]**.** The inhibitory PD-1-PD-L1 interactions are implicated in immune evasion strategies utilized by several pathogens causing chronic infections as was shown initially by studies on Lymphocytic Choriomeningitis virus (LCMV) infection in mice.

Initial studies with bovine leukemia virus (BLV) has shown that mononuclear cells in the peripheral blood from late-stage disease of BLV-infected animals exhibit noticeably more Interleukin-10 (IL-10) mRNA compared to uninfected animals or animals in the early stages of disease. In contrast, IL-2 and IFNγ declines with disease progression [11]. Additionally, T cell functioning in BLV seropositive alymphocytotic (AL) cattle has been compared to T cell functioning in animals with more severe infection. The results showed that when the illness worsened and led to the development of tumors and chronic lymphocytosis, CD4^+^ T cell proliferation was inhibited [12]. Besides compromised CD4^+^T cell proliferation, cytokines such as IL-12 mRNA expression decreased in persistent lymphocytosis [13,14]. All of these signs are consistent with the exhaustion phenotype and in support of this the exhaustion molecule PD-1 was shown to be up-regulated on PBMCs of leukaemic animals as is further discussed later [15]. An increase in PD1^+^ T cells have been reported in BLV infection and its ligands PD-L1, PD-L2 expressed on the surface of dendritic cells or macrophages [16]. The activated T cell surface expresses PD-1 receptors on its surface [17]. The representation of PD-1^+^ CD4^+^ and CD8^+^ T cells in lymph nodes was higher in BLV-positive cattle with lymphoma than it was in BLV-positive cattle without lymphoma or in control uninfected cattle, according to data from PD-1 expression and functional studies on PBMC isolated from BLV-infected cattle [18]. Furthermore, PD-1 inhibition reduced BLV-gp51 expression while increasing the proliferation of PBMCs and their IFNγ production [18].

**Fig. 2:**
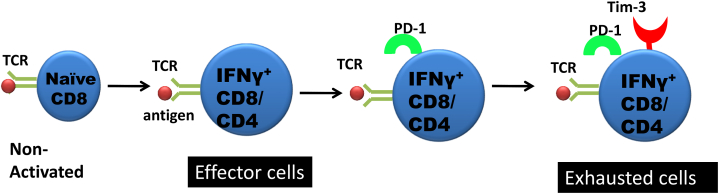
Co-expression of TIM-3 and PD-1 Correlates with a severe exhaustion of immune cells during chronic infections and tumors. TIM-3^+^PD-1^+^ Exhausted T Cell Produces Inhibitory Cytokines.

TIM-3 is a type I *trans*-membrane protein that was initially identified in an attempt to delineate novel cell surface markers that would help in identifying IFNγ producing Th1 and cytotoxic T (Tc1) cells [19]. Galectin-9 (Gal-9), (a ligand for TIM-3) upon binding to TIM-3 leads to intracellular calcium flux, aggregation, hampering proliferation of cells and cytokine secretion as well as T cell death. Classically, TIM-3 is expressed on activated T cells (Th1, CD4^+^ and CD8^+^ cells) and is responsible for selective loss of activated effector T cells and subsequent suppression of effector responses. This indicates a role of the TIM-3/Gal-9 pathway (Fig. 1A) in ensuring effective termination of effector responses [20,21]**.** TIM-3 and its ligand, Gal-9 expression patterns have been characterized using quantitative real-time PCR in cattle following BLV infection. Up regulated TIM-3 mRNA expression in CD4^+^ and CD8^+^ cells with the advancement of BLV infection also has been reported [22]. Positive correlation of TIM-3 and Gal-9 expression levels were associated with the increase of viral load that occurred in BLV- infected cattle. When both TIM-3/Gal-9 and PD-1/PD-L1 pathways were blocked, there was significantly enhanced IFNγ mRNA expression in comparison to the individual pathways alone, suggesting a synergism in mechanisms (Fig. 2) and pointing towards the role of TIM-3 in the down regulation of T cell function in BLV pathogenesis [22].

LAG-3 is a receptor displayed on the surface of T-cells. It demonstrates a greater binding affinity for class II MHC molecules on classical antigen-presenting cells and B cells when compared to the CD4 molecule [23]**.** In a mouse model of LCMV, exhausted cells expressed high levels of LAG-3 [24]. Along with TIM-3 and PD-1, LAG-3 is yet another immuno-inhibitory pathway regulating immune responses following chronic infections and also acts as animmune evasion strategy by several pathogens including those that cause chronic infections. LAG-3 expression was increased and positively correlated with the increase of MHC class II^+^ expression on cells and lymphocytosis following BLV infection [25]. Both, the expression levels of immuno-inhibitory receptors alone or their co-expression with each other on bovine PBMCs have been studied in various chronic diseases. Interestingly the representation of PD-1^+^LAG-3^+^ co-expressing [26] CD4^+^ and CD8^+^ T cells was significantly increased in bovine blood with B-cell lymphoma compared with uninfected animals with uninfected animals as well as in BLV-infected animals but without lymphoma**.**

The generation of effector responses is regulated by a plethora of cellular and molecular events. Besides, immune responses, for example, are subjected to control by CD4^+^CD25^+^ FoxP3^+^ regulatory T cells (Tregs). Several studies have delineated the role of CTLA-4, a Treg-associated marker, in the progression of several diseases [27]. The proportion of CD4^+^FoxP3^+^Tregs correlates positively with the lymphocyte number and viral load but negatively or inversely correlated with IFNγ levels in BLV infected cattle [28].The frequencies and representation of CD4^+^CD25^high^FoxP3^+^ T cells and TGF-β^+^ cells were shown to be increased in response to infection suggesting a role for TGF-β in the dysfunction of CD4^+^ T cells and NK cells [29]**.**This therapeutic strategy warrants further studies on greater number of animals to verify its effectiveness for use in the clinics.

b. ***Anaplasma marginale****:* Antigen specific effector and memory responses and high affinity antibody (IgG) responses are critical for control of blood-borne infections. Another persistent infection of cattle, characterized by acute and chronic high-load bacteremia, is caused by *Anaplasma marginale. A. marginale*-specific CD4^+^ T cell responses are functionally impaired throughout infection as indicated by diminished antigen-specific T cell proliferation and IFNγ producing cells and the absence of long-term memory [30]**.** CD4^+^, CD8^+^, and γδ T-cell populations co-expressing PD-1^+^and LAG-3^+^ T cells progressively increase and peaked at 5 weeks post *Anaplasma* infection. The co-expression of the immuno-inhibitory receptors PD-1 and LAG-3 on T cells resulted in developing the exhausted phenotype during bovine anaplasmosis [26]**.**
c. **Johne's disease**: Production losses in livestock industry are mainly due to chronic illnesses such as Johne's disease which is characterized by persistent granulomatous enteritis, resulting in long-standing diarrhea [31]**.** Clinical cases of diarrhea are treated with classical anti-diarrhoeal agents in the clinics but a significant number of animals do not respond to the treatment and become progressively emaciated [32]**.** Bovine immuno-inhibitory receptors are shown to play crucial role also in the in diminution of *Mycobacterium avium* subspecies *paratuberculosis (*MAP*)*-specific T-cell responses [33]**.** Additionally, the addition of PGE2 therapy led to decreased T-cell proliferation and cytokine release as well as the increased expression of anti-inflammatory molecules on PBMCs in seemingly healthy cattle that included IL-10 and PD-L1. PGE2 levels were found to be enhanced in serum and intestinal lesions of cattle afflicted with Johne's disease. PBMCs from MAP positive cattle when stimulated with Johnin purified protein derivative (J-PPD) resulted in cyclooxygenase-2 (COX-2) transcription, PGE2 generation and increased expression of PD-L1 and immune-inhibitory receptors [34]. These data suggest the mechanism where the state of inflammation following infection induces prostaglandins, which in turn enhances the expression of immune-inhibitory receptors on PBMCs leading to the exhausted phenotype. The Th17 axis is believed to play a role in infections caused by *Mycobacterium*, such as *Mycobacterium tuberculosis* [35]**,** *Mycobacterium bovis* [36]**,** and MAP. Th17 promoting cytokines such as IL-23, IL-22, IL-17 as likely contributors to the development and maintenance of inflammation following MAP infection and its progression to the clinical stage of paratuberculosis [37].
d. **Bovine viral diarrhoea virus** (BVDV): PD-1 and PD-L1 mRNA and the receptor proteins are significantly increased following BVDV infections. This phenomenon was observed in both cytopathic (CP) and non-cytopathic (NCP) BVDV infections (strains NADL and KD). The proliferation potential of peripheral blood lymphocytes (PBLs) were diminished in conjunction with the up-regulation of PD-1/PD-L1 [38].
e. **Fascioliasis:***Fasciola hepatica* establishes chronic infections in bovines even in the presence of strong Th2 responses [39]**.**The anti-inflammatory cytokines IL-10 and TGF-β, act to limit IL-4 and IFNγ production [40]**.**The defective T cell effector responses are characterized by an absence of antigen-specific proliferation and cytokine production and were attributed to T-cell- intrinsic exhaustion within the hepatic lymph node (HLN) where, HLN- CD4 T-cells displayed increased levels of PD-1 expression and diminished levels of IL-2 secretion. Curiously PD-1 expressing cells were not present in the PBL population [41]**.**
f. ***Mycoplasma bovis:*** The generation of effective effector responses are compromised following *Mycoplasma bovis* infection [42]. This could be explained by the increased representation of PD-1^+^CD4^+^ and PD-L1^+^CD14^+^ cells (evidence of exhaustion) in the peripheral blood of *Mycoplasma* positive cattle. Furthermore the numbers of PD-1^+^CD4 and CD8Tcells were correlated negatively with effector responses such as IFNγ production from PBMCs and *in-vitro* inhibition of PD-1/PD-L1 enhanced increased IFNγ production by effector cells [43]**.**The effector cells undergo rapid proliferation upon antigen exposure, but suppressed PBMCs proliferation and diminished antigen specific T cell responses were observed following exposure of cattle to *M. bovis* infection [44]. In yet another study mRNA transcripts of immuno-inhibitory markers such as PD-1, PD-L1, LAG-3, and CTLA-4 were upregulated in mononuclear fractions of milk in *M. bovis* infused mammary gland quarters [45]**.**

The increase of PD-L1^+^ monocytes in *M. bovis*-infected cattle has been attributed to increased plasma PGE2 levels suggesting a role for PGE2 as an inducer of PD-L1 expression. However, of interest, IFNγ production from PBMCs was down regulated in *M. bovis*-infected cattle [46].

Having demonstrated the involvement of the PD-1/PD-L1 pathway and PGE2 in immuno-suppression following *M. bovis* infection, dual blockade of the PD-1/PD-L1 pathway and PGE2 in *M. bovis* infection in vivo was evaluated using anti-bovine PD-L1 rat-bovine chimeric antibody, Boch4G12, and the COX-2 inhibitor, meloxicam. This combinatorial therapy significantly improved *M. bovis-*specific IFNγ responses in calves, whereas the IFNγ response was not elevated either in the control animals (without any therapy) or in cattle treated with meloxicam alone throughout the experimental period [46]**.** Interestingly, bacterial loads in the nasal discharge and broncho-alveolar lavage fluids among calves treated with Boch4G12 with or without meloxicam were significantly decreased. These results suggest that the combination of anti-PD-L1 antibody with a COX-2 inhibitor is a valuable therapeutic for calves with *M. Bovis* infection [46]**.**

Thus the data in bovines have reported T cell exhaustion involving both CD4/CD8T cells in animal diseases.

## 3 There is some evidence of B cell exhaustion in diseases of veterinary importance

B-cell exhaustion has been shown to occur in HIV infection and other diseases characterized by immune activation [47]**.** Exhausted B cells up-regulates inhibitory receptors, proliferate less and generate weaker antibody responses against pathogens [[48], [49], [50]]. In the case of ruminants, disruption of both B- and T-cell immune responses in BLV infected cows could be attributed to the exhaustion phenotype in bovine B cells but this need to be formally shown. Diminished humoral immune responses and aberrant cell-mediated immunity following vaccination in cattle that were infected naturally with bovine leukemia virus has been observed and this might be explained by a state of B cell exhaustion [51]. Altered expression of CD5 on B lymphocytes from MAP infected cattle has been reported [52]. We still lack the sufficient basic knowledge related to bovine immune function perhaps due to a paucity of a tool box to perform the immunological studies required to assess immune function. Thus more innovative research is needed to determine the ideal timing to vaccinate animals with newer vaccines and adjuvants which will permit new developed vaccines and vaccination protocols (such as vaccinating newly transported cattle which may be under stress).

## 4 Experimental detection of T cell exhaustion in bovines

**FACS** (fluorescence-activated cell sorter) single cell analysis of multiple parameters in solution can be facilitated by flow cytometry. In flow cytometers, lasers as light sources are used that produce both scattered and fluorescent light signals which are read by photodiodes and photomultiplier tubes. From these, electronic signals are generated and converted to a standardized format (.fcs) data file.

Assessment of number of lymphocytes and their activation status of the immune cells could be evaluated by the expression of the activation and exhaustion markers by staining with antibodies specific for activation markers (for example CD44^hi^ and CD62L^lo^) and exhaustion markers (for example TIM-3, PD-1, LAG-3) etc [53]. In the case of large animals, blood could be harvested at required time points. Histopaque density gradient is used to isolate PBMCs. 1 × 10^5^/100 μL PBMCs in 100 μl of FACS buffer and 0.1% sodium azide are stained [54]**,** with fluorescently-labelled antibodies against the lymphocyte cell surface markers, the activation markers and exhaustion markers. Unlabeled anti-CD16/CD32 is used to block nonspecific Fc receptor-mediated binding in mice, however due to the non-availability of anti-CD16/CD32 blocking antibodies for cattle, so blocking with serum such as fetal bovine serum (FBS) or goat serum [55] is used instead.1% paraformaldehyde (PBS plus 1% FBS), 0.1% sodium azide (NaN3) is used to fix the cells and acquired on a flow cytometer and FACS data analysis is done by FlowJo software. For example flowcytometry assays can detect antigen-specific CD4 T-cells that up-regulate CTLA-4 and PD-1 following *Fasciola hepatica* infection of ruminants [41]**.**

### 4.1 Evaluation of cytokine production by PBMCs

In ICCS (Intracellular cytokine staining) assay [54], 96-well U-bottom plates, 10^6^ cells are cultivated per well for the ICCS assay**.** Cells are un-stimulated or stimulated and kept at 37 °C in 5% CO_2_ with either antigen or non-specific polyclonal stimulation. Brefeldin A (10 μg/mL) is added to the culture to enhance the accumulation of intracellular cytokines. For intracellular cytokine labeling of IFNγ and cell surface marker staining, utilize the Cytofix/Cytoperm kit (BD Pharmingen). All samples are collected with a Flowcytometer and analysed by FlowJo. The cytokine ELISA is performed on culture supernatants to assess the pro-inflammatory/anti-inflammatory cytokine levels [56,57]. Cyokine production has been evaluated in cattle by the ICCS assay [58]**.**

### 4.2 Detection of recall responses to infections

Frequencies and absolute numbers of IFNγ^+^ TNFα^+^ T cells post challenge are evaluated by FACS analysis [21] in peripheral blood. Similarly recall responses has also been flow cytometrically evaluated in chronic diseases in bovines [59]**.**

### 4.3 Lymphocyte proliferation assays and IFNγ ELISA (IFNγ release assay)

Lympho-proliferation and IFNγ production is stimulated by cognate antigen. Blood samples are collected from animals suspected to have an ongoing infection for testing by the IFNγ release assay. Whole-blood stimulation, 48-well plate is used in which heparinized blood (500 μl per well) is placed and stimulated with antigen. The negative control wells contain only culture medium. The incubation is performed for 48 h at 37 °C in air supplemented with 5% CO_2_, as described previously (**56**). Once the supernatant has been harvested, it can be kept at −20 °C until it is utilized for cytokine ELISA [60]. The IFNγ enzyme-linked immunosorbent assay (ELISA) has been described previously [56]**.** Then carboxy fluorescein diacetate succinimidyl ester (CFSE) dilution assay is yet another assay performed to assess lymphoproliferation [61]**.**

### 4.4 Epigenetic regulation of T cell exhaustion

Various studies have shown that T cell exhaustion also leads to whole scale epigenetic remodeling which in turn renders these cells with phenotypic stability and inhibits T cell impairment by checkpoint blockade [62]. Multiple technologies such as mapping histone modifications and the location of transcription factors, DNA methylation and three-dimensional genome conformation are deployed to profile the epigenomeby multiple platforms [63,64]. A study involving analysis of bovine CD4^+^ T cell methylome concluded that DNA methylation induces differential gene expression signatures in CD4^+^ T cells from *M. bovis* positive cattle. These differential methylation changes proximal to key inflammatory gene loci may be responsible for the development of non-protective CD4^+^ T cell response during mycobacterial infection incattle [64]**.**

## 5 Can we reverse exhaustion and achieve improvement of livestock health?

### 5.1 Blockade of immune-inhibitory interaction

#### 5.1.1 CTLA-4 inhibitors

An immunological inhibitory receptor, which is found on both regulatory and effector T cells that have been activated, is known as CTLA-4 [65]. To keep the immune response's balance, CTLA-4 attaches to CD80 or CD86 and sends out immune-inhibitory signals [66]. Recent research has shown that malignant neoplasms and persistent infections both up regulate CTLA-4, which affects the host immune system. However, using an antibody to prevent CTLA-4 and CD80 or CD86 binding improves the immune response to these diseases [67]**.** Anti-bovine CTLA-4 antibodies are capable of reactivating lymphocyte activities and may be used as a new treatment for chronic illnesses that have failed to respond to current treatments. Future clinical applications will necessitate additional research [68]. Similar kind of study has been performed in dogs where in order to encourage anti-tumor immunity in dogs with malignancies that are immune-responsive, the research has laid the path for the *in-vivo* evaluation of the first entirely canine, anti-canine CTLA-4 antibody [69], a brand-new CTLA-4 inhibitor based on nanobodies to treat cancer patients in dogs [70]**.**

#### 5.1.2 PD-1/PD-L1 inhibitors

Activated T cells display the PD-1 receptor. Dendritic cells and macrophages both express its ligands, PD-L1 and PD-L2. Immune checkpoint proteins like PD-1 and PD-L1/PD-L2 function as co-inhibitory factors that can stifle or restrict the growth of the T cell response. IFNγ production and proliferation were increased by PD-1 inhibition, and BLV-gp51 expression was decreased. Increased T cell activity and decreased bovine leukemia virus expression in B cells both resulted if bovinePD-1 was blocked *in*-*vitro* [18,71]. Similarly increased proliferation of CD4^+^ T cells occurred following the administration of anti-bovine PD-L1 rat monoclonal antibody 4G12 into a BLV-infected cow [72]**,** with the approach having a promising future.

Studies on cell exhaustion in cattle have shown that in cases of infection with the BLV, Johne's disease, and bovine anaplasmosis, immuno-inhibitory molecules, such as PD-1/PD-L1, are seemingly involved for immune exhaustion and disease advancement [73]. It has been reported that BLV infection results in an increase in PD-L1-expressing cells and that PD-L1 inhibition improves *in-vitro* antiviral immune responses [73]. Aside from restoring proliferation and inhibiting apoptosis, PD-1 inhibition also enhanced IFNγ production by stimulated PBL and animals showed reduced BVDV load. After *in-vitro* infection with CP and NCP BVDV, PD-1 inhibition prevents lymphocyte death and restores proliferation and antiviral immune activities [38]**.***Mycoplasma bovis* has been shown to inhibit proliferation of bovine PBMCs. This *Mycoplasma bovis* mediated inhibition is readily ameliorated upon PD-1 Receptor blockade [43]. Furthermore, PD-1/PD-L1 blockade resulted in enhanced IFNγ production [43]. Cattle immunized with OMs were challenged with *A. marginale* after which the frequencies and representation of PD-1 and LAG-3 co-expressing cells in the CD4^+^, CD8^+^, and γδ T-cell fraction were shown to gradually increase. Moreover, when PD-1 and LAG-3 pathways were blocked, the proliferation of OM-specific PBMC and IFNγ secretion was restored to a considerable extent. Thus such studies support the idea that a fraction of T cells expressing PD-1 and LAG-3 represent a population of *A. marginale*-specific exhausted T cells after infection and that suppressed T-cell responses in bovine anaplasmosis seems to be regulated by these immunoinhibitory receptors [26]. CD4 T-cells recovered from the HLN of cattle infected with *Fasciola hepatica* had high levels of PD-1 expression and low levels of IL-2 secretion. When IL-2 was administered, this defect was partially rescued and a combined IL-10 and TGF-β neutralization fully restored the proliferation, and cytokine production by these cells. It still needs to be demonstrated whether such exhausted cells contributes to the usual failure of resistance to re-infection in fascioloasis [41].

In an attempt to develop strategies to reverse immune exhaustion, the use of a COX-2 inhibitor, inhibited production of PGE_2,_ which in turn activates BLV-specific Th1 responses *in-vitro* was investigated. This resulted in increased proliferation of T cell and cytokine secretion by Th1 cells, and decreased viral load of BLV in vivo. A combined approach including the injection of meloxicam (a COX-2 inhibitor) and an antibody against PD-1 led to a considerably reduced BLV virus load, suggesting that it might function as a novel and efficient preventative intervention against BLV infection. It is necessary to conduct additional research using more animals to demonstrate the efficacy and efficiency of this treatment before it can be used in clinical trials. PGE2 therapy reduced T-cell proliferation and Th1 cytokine secretion and increased levels of immuno-inhibitory receptors including IL-10 and PD-L1 in PBMCs of healthy cows during *in-vitro* tests.

### 5.2 Manipulating regulatory T cell activity

Regulatory T cells as the name suggests, regulates effector responses, which could have either a good and favorable or a bad and unfavorable impact depending on context of a specific animal disease. Most of the time the ratio of Tregs to T effector cells is crucial in deciding the outcome of an infection or a disease. Increased Treg responses typically presented in chronic infections can help the host by inhibiting effector T cells and by doing so may reduce the magnitude of immunopathology [74]. But extensive manipulation of an infectious agent by Tregs can lead to impaired survival and long term persistence of the infection. It is even believed that certain pathogens may activate or induce Tregs [75,76], and is thus part of their immune evasion regime. Treg immunotherapy has now become very relevant [77], and depending on the type and pathogenesis of the disease, the therapeutic strategy may be to enhance (for inflammatory diseases) or decrease (for cancer) Tregs [78]**.** The majority of the research related to Tregs is derived from lab animal models or human disease. In veterinary medicine, the literature is still scanty and warrants further investigation. Treg activity may be therapeutically managed. It is clear that the signature CD4^+^CD25^+^FoxP3^+^ cells naturally exist in cattle [79]. However, evidences suggests that in cattle the CD4^+^CD25^high^Foxp3^+^ and CD4^+^CD25^low^ T cells do not function as Treg *ex-vivo*. The bovine Treg function is vested inγδT cell population, more precisely in the WC1.1^+^ and the WC1.2^+^ subpopulation, which are the major cells in cattle blood in contrast to non-ruminant species [80]. Research must now move to examine the actual function of potential bovine Treg cells. Treg associated cytokine TGFβ and transcription factor FoxP3 were unaffected by the progesterone levels in the pregnant cows [81]. The evaluation of mRNA transcript associated with Tregs (CD25, FoxP3, CTLA-4, and IDO), after experimental infection of beef calves with low (LV) or high (HV) virulence BVDV have also been studied. In order to explain if immuno-suppression caused by BVDV could be associated with regulatory T lymphocyte activity [82].

Numerous cellular and molecular processes, such as Treg that are CD4^+^CD25^+^, regulate the immune system and the development of T cell immunity. Recent research revealed that the development of numerous diseases is closely correlated with the presence of the Treg-associated marker CTLA-4. It has been observed that in cattle infected with the bovine leukemia virus, the fraction of FoxP3^+^CD4^+^ cells was inversely connected with IFNγ expression but positively correlated with the lymphocyte number, virus titer, and virus load [83]**]**. Moreover CTLA-4 mRNA was predominantly expressed in CD4^+^ T cells in BLV-infected cattle, and the expression positively correlated with Foxp3 mRNA expression [17]. MAP antigen stimulation resulted in IL-10 production and that neutralization of this IL-10 enhanced IFNγ production from MAP-antigen specific effector T cells. Depletion studies revealed that IL-10 producing cells were largely CD4^+^ and CD25^+^ [84]**.** Populations of CD4^+^CD8^+^FoxP3^+^ Tregs have been shown to develop in response to low level antigenic stimulation with MAP [85], moreover, the number of FoxP3^+^ cells increased in parallel with the increased severity of intestinal lesions [86]. Bovine leukemia virus reduces anti-viral cytokine activities and NK cytotoxicity by inducing TGFβ secretion from regulatory T cells [29]**.** These observations suggest that higher Treg activity correlates with diminished pathogen specific immune responses and that manipulating Treg activity may achieve favorable outcomes.

### 5.3 Cytokine therapy

Cytokines modulate both innate and adaptive immune responses by allowing cells of the immune system to communicate with each other both in a paracrine and autocrine manner [87]. Furthermore, they control leucocyte survival, effector functions, and immune cell proliferation and differentiation. The majority of studies with cytokines are performed either *in-vitro* or performed or in mice or in other lab animal model systems. There are challenges associated with the use of cytokines in-vivo mainly due to the issues related to redundancy and pleiotropism and in ruminants the exorbitant cost involved. Prior infection history in clinical cases may also influence the outcome of cytokine therapy. For example TNF is required for naive mice to control vaccinia virus infection [88]. However, with previous LCMV infection, the mice do not need TNF for efficient vaccinia virus clearance. Thus, anti-TNF therapies required for treatment of various diseases may be safe in animals with prior LCMV infection compared to the naive animals. Finally more detailed understanding on use of cytokines in ruminants [[89], [90], [91]] is still crucial for the development of new immuno-therapies to facilitate the reversal of immune exhaustion.

## 6 Concluding remarks and open questions

In this review we discussed the occurrence of immune exhaustion in various infectious diseases of ruminants. Among the innate and adaptive immune defenses, T cells of the adaptive immune arm display a myriad of characteristic features that qualify the cells to be assigned as exhausted. However, some evidence of B cell exhaustion does exist but there is minimal evidence that exhaustion affects the innate immune participants. Immune exhaustion if predicted could be confirmed by a plethora of techniques that are discussed in detail. Immune exhaustion has been shown to occur in chronic infections of ruminants such as paratuberculosis, anaplasmosis, BLV, BVDV, parasitic infections and many more. In such conditions the animals do not respond to the classical therapeutic strategies used and the health status deteriorates. In this context, it is thus crucial to delineate whether exhaustion occurs and to explore the use of novel therapeutic strategies to reverse the phenomenon. These could include therapies [92], blocking immuno-inhibitory interaction to augment immunity [21], cytokine therapy, manipulating Treg activity, and reversing the state of exhaustion. All of these approaches represent a paradigm shift in the management of chronic infections in bovines. Answering such questions may transform therapeutic modalities for chronic and persistent infections in ruminants. Currently, the literature available on ruminant immunology is very scanty and the majority of studies are done in experimental infection models which do not translate well to the natural clinical setting where huge individual variations in immune responses exist from animal to animal, possibly due to heterologous immunity [[92], [93], [94]] and individual infection history of the animal. Overall we are optimistic that checkpoint blockade therapy will have a place in managing some bovine diseases but for the economic reasons its use will be limited.

## Funding

This work was supported by 10.13039/100019566National Agricultural Science Funds (NASF) grant to Shalini Sharma (grant NASF/ABS-9009/2022–23) and UTCVM Center of Excellence program, 10.13039/100014455UT Knoxville, TN, USA.

## Institutional review board statement

Not applicable.

## Informed consent statement

Not applicable.

## Data availability statement

Not applicable.

## CRediT authorship contribution statement

**Shalini Sharma:** Writing – review & editing, Writing – original draft, Supervision, Investigation, Funding acquisition, Formal analysis, Data curation, Conceptualization. **Naveen Kumar:** Writing – original draft, Resources, Conceptualization. **Barry T. Rouse:** Writing – review & editing, Writing – original draft, Methodology, Investigation, Formal analysis, Conceptualization. **Khushbu Sharma:** Writing – review & editing, Writing – original draft, Investigation, Conceptualization. **Kundan Kumar Chaubey:** Formal analysis. **ShoorVir Singh:** Investigation, Data curation, Conceptualization. **Praveen Kumar:** Investigation. **Pradeep Kumar:** Investigation.

## Declaration of competing interest

The authors declare no conflict of interest.
